# The risk of venous thromboembolism in RA

**DOI:** 10.1093/rheumatology/keag388

**Published:** 2026-07-25

**Authors:** Tamara Vojinovic, Johan Askling, Aleksandra Antovic

**Affiliations:** Division of Rheumatology, Department of Medicine Solna, Karolinska Institutet, Karolinska University Hospital, Stockholm, Sweden; Division of Clinical Epidemiology, Department of Medicine Solna, Karolinska Institutet, Stockholm, Sweden; Rheumatology, Theme Inflammation and Ageing, Karolinska University Hospital, Stockholm, Sweden; Division of Rheumatology, Department of Medicine Solna, Karolinska Institutet, Karolinska University Hospital, Stockholm, Sweden; Rheumatology, Theme Inflammation and Ageing, Karolinska University Hospital, Stockholm, Sweden

**Keywords:** RA, venous thromboembolism, systemic inflammation, endothelial dysfunction, haemostatic balance

## Abstract

RA is associated with a markedly increased risk of venous thromboembolism (VTE), reflecting a complex interplay between chronic inflammation, immune dysregulation and haemostatic imbalance. Large population-based studies consistently demonstrate a 50–100% excess risk of deep vein thrombosis and pulmonary embolism in RA, with the highest incidence early after diagnosis and during flares. Mechanistically, inflammatory cytokines, endothelial dysfunction, platelet activation, impaired fibrinolysis and autoantibody-driven immune responses promote a state of chronic immunothrombosis. RA-specific factors such as disease activity, seropositivity, disability and treatment exposures further modify thrombotic risk; some targeted therapies may amplify the risk in a subset of patients. Despite these insights, current VTE risk stratification and prevention strategies are extrapolated from the general population and fail to incorporate RA-specific factors. Improved understanding of the reason(s) behind the increased VTE risks reported with certain immune-modulatory drugs, and development of integrated clinical and biomarker-based stratification tools, are therefore both essential to enable effective thromboprophylaxis in RA.

Rheumatology key messages

- RA is associated with a significantly increased risk of venous thromboembolism (VTE).
- Inflammation, immune dysregulation, disease activity and treatments contribute to thrombotic risk in RA.
- Better risk stratification, prevention and mechanistic understanding are needed to improve VTE management in RA.

## Introduction

RA is associated with a markedly increased risk of venous thromboembolism (VTE), the third most common vascular event in patients with RA encompassing both deep vein thrombosis (DVT) and pulmonary embolism (PE), compared with the general population [1]. The increased risk of VTE in RA is not isolated but rather part of a spectrum of cardiovascular risks including (to varying degrees) atherosclerotic cardiovascular disease (CVD), acute ischaemic events, atrial fibrillation, heart failure and both ischaemic and haemorrhagic cerebrovascular disease [2, 3].

In this review, we provide a comprehensive overview of the epidemiology, risk factors and pathophysiological mechanisms underlying VTE in RA. We synthesize evidence from population-based studies and mechanistic research to describe the contribution of systemic inflammation, immune dysregulation and treatment-related factors to thrombotic risk. In addition, we discuss the impact of RA disease activity and antirheumatic therapies on VTE risk, current approaches to prevention and management and key knowledge gaps that warrant future investigation.

## Occurrence

Evidence from large population-based cohort studies and meta-analyses consistently demonstrates a 50–100% increased risk of VTE among individuals with RA, with reported adjusted hazard or odds ratios ranging from ∼1.3 to 2.2, independent of traditional VTE risk factors such as age, sex, BMI and exposure to oestrogen-containing contraceptives or hormone replacement therapy [1, 3–7]. The absolute incidence of VTE in RA is estimated at 3–6 events per 1000 person-years, with the highest risk observed during the first year after diagnosis, after which it gradually declines but remains elevated long-term [3, 7, 8] (Table 1).

**Table 1:** Seminal observational studies assessing the risk of VTE in RA.

Author	Design	Country	Year	Size and events	Outcome	Incidence	Relative risk
Holmqvist *et al.* [3]	Cohort	Sweden	1997–2010	37 856 RA patients 838 VTE events	VTE	Incidence rate VTE: 5.9/1000 PYs	HR (95% CI) VTE: 2.0 (1.9–2.2)
Kim *et al.* [4]	Cohort	USA	2001–2008	22 143 RA patients 265 VTE events	Overall and DVT + PE separately	Incidence rate VTE: 6.1/1000 PYs PE: 2.6/1000 PYs DVT: 4.5/1000 PYs	HR (95% CI) VTE: 1.4 (1.1–1.7) PE: 1.9 (1.3–2.7) DVT: 1.2 (0.9–1.5)
Choi *et al.* [5]	Cohort	UK	1986–2010	9 589 RA patients 82 PE events 110 DVT events	Overall and DVT + PE separately	Incidence rate PE: 1.5/1000 PYs DVT: 2.01/1000 PYs	RR (95% CI) PE: 2.16 (1.68–2.79) DVT: 2.16 (1.74–2.69)
Ogdie *et al.* [6]	Cohort	UK	1994–2014	51 762 RA patients 1 479 VTE events	Overall and DVT + PE separately	Incidence rate VTE: 7.9/1000 PYs PE: 2.0/1000 PYs DVT: 6.2/1000 PYs	HR (95% CI) VTE: 1.35 (1.27–1.44) PE: 1.74 (1.55–1.99) DVT: 1.29 (1.20–1.38)
Li *et al.* [8]	Cohort	Canada	1997–2015	39 142 RA patients 1 432 VTE events	Overall and DVT + PE separately	Incidence rate VTE: 3.79/1000 PYs PE: 1.43/1000 PYs DVT: 2.82/1000 PYs	HR (95% CI) VTE: 1.28 (1.20–1.36) PE: 1.25 (1.13–1.39) DVT: 1.30 (1.21–1.40)
Russell *et al.* [9]	Cohort	UK	1999–2018	23 410 RA patients 845 VTE events	VTE	Incidence rate VTE: 4.42/1000 PYs	HR (95% CI) VTE: 1.46 (1.36–1.56)
Conforti *et al.* [10]	Cohort	Italy	2010–2020	347 RA patients 13 VTE events	VTE	Incidence rate VTE: 7.8/1000 PYs	–
Molander *et al.* [11]	Cohort	Sweden	2006–2018	46 316 RA patients 2 241 VTE events	VTE	–	HR (95% CI) VTE: 1.88 (1.65–2.15)
Kang *et al.* [12]	Case-control	Taiwan	2001–2009	5 193 DVT cases 20 772 controls 245 RA patients	DVT	–	OR (95% CI) VTE 1.88 (1.42–2.58)
Chung *et al.* [13]	Cohort	Taiwan	1998–2008	29 238 RA patients 278 VTE events	DVT and PE separately	Incidence rate PE: 0.36/1000 PYs DVT 1.01/1000 PYs	HR (95% CI) PE: 2.07 (1.55–2.76) DVT: 3.36 (2.79–4.03)
Matta *et al.* [14]	Cohort	USA	1979–2005	4 818 000 patients 41 000 PE events 79 000 DVT events	Overall and DVT + PE separately	PE: 41 000/4 818 000 (0.85%) DVT: 79 000/4 818 000 (1.64%)	RR (95% CI) VTE: 1.99 (1.98–2.00) PE: 2.25 (2.23–2.27) DVT: 1.90 (1.89–1.92)
Bacani *et al.* [15]	Cohort	USA	1980–2007	813 RA patients 19 VTE events	VTE	Cumulative incidence 6.7 vs 2.8 for non-RA subjects	–

In RA and other systemic autoimmune diseases, systemic inflammation represents a shared risk factor for VTE although the magnitude of risk increase varies between diseases. The risk of VTE is higher in patients with RA than in those with psoriatic arthritis, with or without severe psoriasis, while SLE and SSc confer an even greater risk compared with RA [6, 16–19].

## Traditional risk factors for VTE

Traditional VTE risk factors such as age, obesity and smoking are also relevant in RA [9, 10]. Although the relative excess risk of VTE due to RA is highest among younger individuals (among whom the absolute incidence of VTE is lower than among the elderly) and among those with normal BMI the absolute (and absolute excess) risk associated with RA increases with increasing age and BMI [10]. Smoking is a recognized risk factor for both CVD and VTE and is prevalent in RA populations [20].

Cardiovascular risk factors, such as diabetes, are independently associated with increased VTE risk in RA. Hospitalization and immobilization, particularly in the context of surgery or acute illness, further amplify this risk. A history of prior VTE, however, remains the strongest predictor of VTE (recurrence) [7, 21].

Pregnancy is a well-established hypercoagulable state that increases the risk of VTE, and while this risk has been extensively documented in autoimmune conditions such as antiphospholipid syndrome and SLE, evidence regarding thrombotic risk in women with RA during pregnancy remains limited [22–25]. Compared with the general population, women with RA face higher risks of preeclampsia, preterm birth and caesarean delivery [26–28]. Moreover, population-based studies suggest that pregnant women with autoimmune diseases, including RA, are at higher risk of pregnancy-related VTE compared with women without autoimmune conditions, underscoring the need for individualized risk assessment and consideration of thromboprophylaxis when appropriate [24, 25].

## RA-specific risk factors for VTE

RA-specific risk factors for VTE include both disease-related and treatment-related factors, as well as comorbidities and demographic characteristics that are particularly relevant in the RA population.

### RA disease activity

The risk of VTE in RA is strongly modulated by the level of disease activity. Large population-based cohort studies demonstrate that patients with higher disease activity, as measured by validated composite scores such as DAS28-ESR or DAS28-CRP, have a substantially higher risk of developing VTE than those in remission or with low disease activity [11, 29].

Specifically, higher RA disease activity, as measured by DAS28-ESR, is associated with an approximately 2-fold increase in VTE risk compared with remission, and persistent elevations in disease activity over time further amplify this risk. Importantly, even patients in DAS28-defined remission remain at an elevated risk of VTE (around 35%) relative to the general population, presumably reflecting the accumulation of other risk factors (e.g. smoking) among patients with RA [11, 29].

A UK population-based cohort study using The Health Improvement Network (THIN) database examined VTE risk among patients with incident RA treated with DMARDs and observed that the risk of PE and DVT was highest during the first year following RA diagnosis, declining steadily over time [5]. This temporal association is consistent with elevated thrombotic risk during periods of active, uncontrolled inflammation early in the disease course. Still, the association between RA disease activity and VTE risk has been insufficiently examined in observational studies due to the limited availability of large-scale data incorporating validated measures of disease activity.

### Autoimmunity

In addition to clinical measures of disease activity, immunological features of RA, particularly seropositivity for RF and anti-CCP antibodies, have been linked to an increased risk of VTE. A large Swedish cohort study of patients newly diagnosed with RA found an increased VTE risk among IgG anti-CCP2-positive patients (hazard ratio (HR) 1.33, (95% CI 1.00–1.78) vs anti-CCP2-negative), and an increased VTE risk for IgM RF positive patients (HR 1.38 (95% CI 1.04–1.82) vs IgM RF negative patients) [30]. These associations were independent of traditional RA risk factors such as smoking and HLA-DRB1 shared epitope alleles. In contrast, antibodies directed against other post-translational protein modifications, such as carbamylation or acetylation, were not associated with VTE risk. However, given the substantial overlap and limited specificity among ACPA specificities and other anti-modified protein antibodies (AMPAs), these findings may reflect a broader autoantibody-positive phenotype rather than strict specificity to citrulline-directed autoimmunity [30].

These immunological associations should also be interpreted in the context of disease activity. Seropositive RA is typically characterized by a more severe and persistent inflammatory phenotype, which is itself a strong determinant of VTE risk. Consistent with this, population-based data indicate that although overall VTE risk in RA is elevated compared with the general population, stratification by RF status alone does not fully explain this excess risk [3]. This suggests that autoantibody positivity, particularly anti-CCP positivity and broader ACPA reactivity, may primarily identify patients with higher inflammatory disease activity, rather than constituting an independent thrombotic risk factor [3, 30].

The contribution of aPLs to VTE risk in RA remains less well defined. The reported prevalence of aPL positivity varies widely (5–75%) across studies due to differences in antibody types, assays and cut-off values, with an overall mean prevalence of 28% and a median of 22% [31]. While antiphospholipid syndrome is a well-established risk factor for VTE, available studies in RA populations have not demonstrated a consistent association between aPL positivity and thrombotic events or other clinical manifestations of APS, likely reflecting limited study size and the lack of standardized APS assessment rather than the absence of risk [31].

Importantly, the interpretation of aPL in RA is further complicated by evolving classification frameworks. The recently introduced 2023 ACR/EULAR APS classification criteria provide a more specific and weighted approach to defining APS, integrating both clinical and laboratory domains to improve specificity and cohort homogeneity. As discussed by Erkan [32], these updated criteria may help address prior limitations in APS research by enabling more standardized identification of patients and facilitating more consistent risk stratification across studies. Their application in RA populations may therefore refine future assessments of the relationship between aPL and VTE risk and help bridge existing gaps in risk modelling.

## Pathophysiological mechanisms

The pathophysiology of VTE in RA is multifactorial, arising from the interplay between disease-specific mechanisms and traditional thrombotic risk factors. Systemic inflammation plays a central role, promoting endothelial dysfunction, activation of the coagulation cascade and a hypercoagulable state. These processes can be conceptually framed within Virchow’s triad of vascular injury, hypercoagulability and venous stasis, of which inflammation in RA predominantly affects the first two components. Thrombotic risk is further amplified by general factors such as advanced age, obesity and immobility, as well as RA-specific determinants including high disease activity and cumulative disease burden, reflected by prolonged disease duration, recurrent inflammation, functional impairment and treatment exposure.

### Endothelial dysfunction

Endothelial injury represents one of the central mechanisms linking inflammation in RA to vascular disease and thrombosis. Impaired endothelial function has been demonstrated early in the disease course, reflecting a persistent proinflammatory state characterized by endothelial activation, increased vascular permeability and enhanced leucocyte and platelet adhesion [33, 34]. Proinflammatory mediators including IL-1β, TNF-α, IL-6 and CRP, promote the expression of endothelial adhesion molecules and tissue factor (TF), thereby facilitating monocyte–endothelial interactions and creating a prothrombotic endothelial phenotype. In parallel, upregulation of endothelial-derived coagulation factors such as von Willebrand factor (vWF) and plasminogen activator inhibitor-1 (PAI-1) contributes to impaired fibrinolysis and increased thrombotic risk [35, 36].

Microvascular endothelial damage occurs very early in the disease course, coinciding with disease onset. However, measurable functional endothelial dysfunction appears to develop over subsequent months, becoming detectable in early RA and consistently present in established disease [34]. This temporal dissociation suggests that endothelial injury precedes overt dysfunction, with sustained inflammatory exposure required to induce clinically relevant impairment of endothelial function.

Importantly, endothelial dysfunction in RA reflects both acute inflammatory activity and the cumulative burden of chronic inflammation. While active systemic inflammation appears to transiently amplify prothrombotic endothelial changes during periods of disease activity or flares, prolonged exposure to inflammatory mediators induces more sustained endothelial alterations [34]. This suggests that both concurrent inflammation and a history of persistent disease activity contribute to an increased VTE risk.

### Immunothrombosis and inflammation

**Figure 1:**
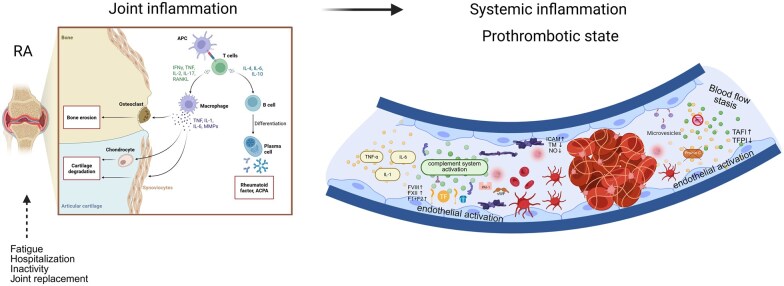
Schematic presentation of immunothrombosis and thromboinflammation mechanisms in RA. Local joint inflammation in RA (left) leads to systemic inflammation, which promotes endothelial dysfunction, activation of coagulation pathways and impaired fibrinolysis within the vasculature (right), thereby creating a prothrombotic state that increases the risk of VTE. Proinflammatory cytochines: TNF-α, IL-1, IL-6, TF, tissue factor; FVIII, factor VIII; FXII, factor XII; F1 + F2, prothrombin fragments 1 + 2; PAI-1, plasminogen activator inhibitor-1; vW, Von Willebrand factor; ICAM-1, intercellular adhesion molecule 1; TM, thrombomodulin; NO, nitric oxide; AT, antithrombin; TAFI, thrombin-activatable fibrinolysis inhibitor; TFPI, tissue factor pathway inhibitor. Created in BioRender. Vojinovic, T. (2026) https://BioRender.com/420rjxv

Chronic inflammation profoundly modulates thrombotic responses by shifting the haemostatic balance towards a prothrombotic state through the upregulation of coagulant pathways and suppression of anticoagulant and fibrinolytic mechanisms [37, 38]. Moreover, activation of the extrinsic coagulation cascade and impairment of fibrinolysis may not only promote thrombosis but also amplify inflammatory responses, creating a self-sustaining cycle of inflammation and hypercoagulability [38]. A key mediator of inflammation-driven coagulation activation is TF, the principal initiator of the extrinsic coagulation pathway. Inflammatory stimuli, such as CRP, proinflammatory cytokines (TNF-α, IL-1 and IL-6) and complement activation, induce TF expression in intravascular cells, including monocytes, endothelial cells and platelets [39, 40]. Elevated circulating TF levels have been observed in patients with RA, particularly in those with active disease [41]. In parallel, RA is associated with increased plasma concentrations of other coagulation factors and thrombin generation markers, including fibrinogen, von Willebrand factor (vWF), factor VIII, activated factor XII, prothrombin fragments 1 + 2 (F1 + 2) and thrombin–antithrombin complexes [37, 42, 43] (Fig. 1).

Additionally, dysregulation of the fibrinolytic system further contributes to a prothrombotic setting in RA. Elevated levels of fibrinogen, tissue plasminogen activator antigen (t-PA), PAI-1, D-dimer and thrombin-activatable fibrinolysis inhibitor have been documented, particularly in patients with high inflammatory burden [37, 44]. TNF-α appears to play a central role in modulating the expression of key components of the fibrinolytic pathway, reinforcing the link between inflammation and impaired clot lysis [37]. While TNF inhibition may favourably influence coagulation and fibrinolytic markers, the overall thrombotic risk in RA is primarily driven by patient-specific factors, rather than by drug-specific effects on the fibrinolytic pathway alone.

Finally, endogenous anticoagulant systems, including antithrombin, TF pathway inhibitor (TFPI) and the protein C system, are downregulated by inflammation. Both TFPI and the protein C pathway exert protective effects on endothelial function, suggesting that their suppression may further amplify thrombotic risk in RA [45, 46]. However, the clinical significance of these haemostatic alterations remains incompletely understood, and prospective evidence linking specific markers to thromboembolic outcomes in RA is still limited. Yet, effective antirheumatic treatments during the first 24 weeks after diagnosis in patients with newly diagnosed RA improved the haemostatic imbalance, with particularly prominent effects of biological drugs, especially tocilizumab, compared with csDMARDs [47].

### The role of platelets and platelet-derived extracellular vesicles

Platelets play an important role in the pathogenesis of RA, extending beyond haemostasis to active participation in inflammatory and thrombotic processes. A key mechanism underlying these effects involves the release of platelet-derived extracellular vesicles (PEVs), generated through plasma membrane shedding upon platelet activation [48]. Elevated circulating PEV levels have been consistently reported in patients with RA and correlate positively with disease activity [49–52]. Within the synovial compartment, PEVs amplify inflammation by stimulating synoviocytes to release proinflammatory cytokines. In parallel, platelet-derived microparticles (PMPs) promote leucocyte–leucocyte interactions and provide a potent procoagulant surface through the exposure of negatively charged phospholipids, facilitating assembly of the prothrombinase complex and localized thrombin generation. Thrombin formed on the PEV surface further enhances inflammatory signalling and platelet activation, inducing additional EV release and establishing an inflammatory–prothrombotic feedback loop [53].

The inflammatory capacity of PEVs is further supported by their ability to activate neutrophils and by evidence that their formation can be triggered by complement activation, a pathway also implicated in RA pathophysiology [53].

PEVs have emerged as important mediators of intercellular communication by transporting a number of bioactive molecules, including cytokines, growth factors, coagulation proteins and regulatory microRNAs, thereby influencing immune responses and tissue remodeling [54]. A recent study demonstrated that PEVs from the synovial fluid of RA patients can travel via the lymphatic system, suggesting a role in disseminating inflammatory signals beyond the joint microenvironment [55]. Observations of PEVs within lymphatic vessels in experimental models of atherosclerosis further support their involvement in vascular inflammatory conditions and atherothrombosis [54].

### Venous stasis and hyperviscosity

Venous stasis and alterations in blood viscosity represent important, though less frequently emphasized, contributors to VTE in RA. Chronic systemic inflammation in RA is associated with increased blood viscosity, largely driven by elevated acute-phase reactants such as fibrinogen, immunoglobulins (especially in the setting of polyclonal gammopathy and high concentrations of RF) and other plasma proteins, which impair blood flow and promote erythrocyte aggregation [33, 36]. ESR reflects some of these aggregation-dependent effects and serves as an indirect measure of inflammation-associated changes that promote increased blood viscosity and venous stasis [33, 36].

Venous stasis in RA may be further amplified by disease-related factors, including reduced mobility due to pain, joint damage and disability, particularly during periods of high disease activity or hospitalization. Immobilization, even when partial or transient, synergizes with inflammation-induced hypercoagulability and endothelial dysfunction to fulfil the venous stasis component of Virchow’s triad. Moreover, age and comorbidities such as obesity and CVD can further impair venous return and exacerbate venous stasis [33, 34, 36].

## RA treatment and VTE risk

The effect of RA treatments on VTE risk is complex and must be interpreted in the context of underlying disease activity and patient comorbidity. Systemic corticosteroid use is known to be associated with an increased risk of VTE in RA, although—as for most of the purported untoward effects of corticosteroid treatment—separating drug effects from confounding by disease activity remains challenging [56]. Observational studies in both the general population and RA cohorts have demonstrated that oral glucocorticoid exposure is associated with a higher risk of VTE, especially during periods of current or recent use and at higher doses [3, 56]. In RA-specific analyses, corticosteroid use has been identified as an independent risk factor for VTE after adjustment for age, comorbidity and other clinical factors, suggesting that glucocorticoids may contribute to thrombotic risk beyond their role as ‘markers’ of active disease (i.e. beyond confounding by indication) [29, 57]. Importantly, since corticosteroid use and its dosing frequently coincide with periods of high inflammatory disease activity, a strong determinant of VTE risk, there is a possibility of additive effects. Available evidence underscores the importance of minimizing the dose and duration of treatment where possible and incorporating corticosteroid use into VTE risk stratification, particularly in patients with additional thrombotic risk factors [56, 57].

Conventional synthetic DMARDs (csDMARDs), including MTX, have not consistently been associated with an increased risk of VTE, and effective suppression of systemic inflammation may indirectly moderate thrombotic risk [3, 11]. Likewise, no direct evidence links leflunomide to an increased VTE risk in RA. VTE is not listed among adverse events in its regulatory safety profile, and although leflunomide may induce hypertension, a recognized VTE risk factor, there are no data demonstrating an associated increase in thromboembolic events [58].

Similarly, large observational studies and registry-based analyses have not demonstrated an excess VTE risk with biologic DMARDs targeting TNF, with some studies reporting comparable or lower VTE rates than among csDMARD-treated patients, the latter raising the possibility of intrinsic effects of TNFi on VTE risk beyond improved RA disease control [59–61]. At present, the safety profile of TNF inhibitors is well characterized and includes risks such as infections, haematologic abnormalities, neurological complications and lymphoid malignancies, but VTE has not emerged as a consistent safety signal for this drug class [62]. For bDMARDs other than TNFi, although individual studies have reported signals of increased risk for individual drugs (e.g. vs TNFi), there is so far no consistent pattern of increased VTE risk with rituximab, abatacept or tocilizumab [63–65].

Likewise, there is currently no evidence that VTE risk differs between biosimilars and their originator biologics, although VTE-specific comparative data remain sparse and most available studies address overall safety rather than thromboembolic outcomes [66]. Overall, for biologic therapies other than JAK inhibitors, differences in observed VTE risk are difficult to disentangle from confounding by indication, treatment channelling and underlying RA disease activity.

Targeted synthetic DMARDs (tsDMARDs), particularly Janus kinase inhibitors (JAKi), represent a more recent therapeutic class used in RA, especially in patients with persistent disease activity despite csDMARDs, a treatment indication shared with TNF inhibitors [67, 68]. In contrast to TNF inhibitors, concerns regarding VTE risk have been raised for JAKi following the ORAL Surveillance post-authorization safety trial, which demonstrated a dose-dependent increase in VTE, primarily PE, in patients treated with tofacitinib compared with etanercept or adalimumab [69]. These findings prompted regulatory agencies to issue warnings and restrict JAKi use in patients aged ≥65 years and in those with prior VTE or CVD, malignancy or other established risk factors.

The evidence from observational studies regarding VTE risk with JAKi is heterogeneous. Collectively, large observational studies have not demonstrated a consistent increase in VTE risk among patients with RA treated with JAKi. In a large US population-based cohort study using administrative claims data, Desai *et al.* found no statistically significant difference in VTE risk between patients initiating tofacitinib and those receiving TNFi, with VTE events remaining infrequent across more than 87 000 patients [67]. Similarly, a French nationwide cohort study including 15 835 RA patients initiating JAKi or adalimumab demonstrated no increased risk of VTE associated with JAKi after adjusting for confounders [70]. Consistent results were observed in a nationwide Korean cohort study by Song *et al.*, in which VTE events were rare and no significant increase in VTE risk was detected among JAKi users compared with TNFi-treated patients [71]. In addition, long-term data from a large US RA registry showed low and comparable VTE incidence rates over 5 years among patients treated with tofacitinib and biological DMARDs [72].

Notably, an exception to these largely concordant findings comes from a multi-database observational study by Salinas *et al.*, which reported an increased VTE risk associated with baricitinib compared with TNFi, an effect driven largely by data from a Swedish registry and from the French National Health Data System (SNDS) [73]. In line with this, Molander *et al.* conducted a nationwide Swedish register-based cohort study, using largely the same Swedish registry data, and observed a 50–100% higher VTE risk among RA patients treated with JAKi (baricitinib and tofacitinib) compared with TNF inhibitor-treated patients. In that study, the VTE incidence in the JAKi cohort was approximately twice that of the overall RA population (all patients with RA, including those not treated with any b/ts DMARD) and 3-fold that of the general population [74]. The inconsistency across observational studies likely reflects methodological heterogeneity, including potential effects from residual confounding for disease activity and comorbidities, short follow-up, low event rates, varying VTE definitions and geographical differences in baseline thrombotic risk.

Given that many cytokines and growth factors are known to have either pro- or antithrombotic effects, and that JAKi interfere with signalling through overlapping JAK–STAT receptor complexes, estimating the net impact of JAK inhibition on thrombotic risk is inherently complex. Consequently, it remains uncertain whether an increase in VTE risk reflects a class effect or is restricted to specific drug with a particular selectivity. While ORAL surveillance provides evidence of increased VTE risk with tofacitinib, findings from the nationwide Swedish cohort study by Molander *et al.* suggest that this risk may not be confined to a single drug, with similar risk patterns observed for tofacitinib and baricitinib, both of which are non-selective JAK inhibitors [74, 75]. Overall, currently, any association between JAKi and VTE risk is complex and remains incompletely defined. Consequently, the magnitude of any increased VTE risk with their use in routine clinical practice, as well as its attribution to JAK inhibition *vs* patient- or disease-related factors, remains uncertain even if groups of patients at elevated risk have been identified. The remaining uncertainty underscores the importance of individualized benefit–risk assessment when selecting antirheumatic therapy, particularly in patients with established VTE risk factors. In this respect, it may be pertinent to recall that risk counselling should be based on the absolute (e.g. numbers needed to treat) rather than relative (e.g. hazard ratio) risk scale, and that risk–benefit assessments are rarely as ‘clean’ as a balance of only one benefit *vs* only one potential harm but instead require a more holistic approach.

## Primary prevention and treatment for VTE

Primary prevention and treatment of VTE in RA patients should follow general VTE principles while accounting for RA-specific risk factors, particularly disease activity, treatment exposures and comorbidities. As no RA-specific thromboprophylaxis guidelines exist, current strategies are largely extrapolated from recommendations for the general population. Primary pharmacological prevention with low-dose low-molecular-weight heparin (LMWH) is recommended during periods of strong transient risk, such as major orthopaedic surgery, fractures requiring immobilization or severe immobilization during hospitalization [76]. In patients undergoing hip or knee replacement, LMWH or direct oral anticoagulants (DOACs) such as rivaroxaban or apixaban are effective and safe, with extended prophylaxis for up to 35 days recommended in those with additional risk factors [77–79]. In acutely ill hospitalized RA patients, thromboprophylaxis should be considered when VTE risk outweighs bleeding risk, whereas routine prophylaxis is not indicated in ambulatory patients without additional risk factors [78, 79].

Pharmacological treatment of established VTE in RA follows standard anticoagulation strategies using either LMWH, warfarin or DOACs, of which the latter has become the first-line treatment for most patients in recent years. Treatment duration is determined by thrombus location and provoking factors, typically 3–6 months for DVT and 6–12 months for PE, with extended anticoagulation considered in recurrent or persistent-risk cases. Thrombolysis, embolectomy or inferior vena cava filters are reserved for selected high-risk situations [80].

Given the chronic prothrombotic state associated with RA, extended anticoagulation may be appropriate in selected patients with ongoing high disease activity, prior VTE, aPLs or continued exposure to prothrombotic therapies such as systemic glucocorticoids or JAKi [3, 11, 29, 81]. In contrast, patients in sustained remission generally exhibit a lower thrombotic risk, and anticoagulation decisions in this setting should be driven primarily by traditional and patient-specific risk factors rather than RA alone. Importantly, effective control of RA disease activity should be considered an integral component of VTE prevention, as suppression of inflammation may reduce thrombotic risk while limiting the need for corticosteroids [11]. Overall, VTE prevention and management in RA should adopt an individualized, risk-based approach integrating traditional and RA-specific risk factors.

## Knowledge gaps and future research

Despite robust epidemiological and mechanistic evidence linking RA to VTE, its translation into clinical risk stratification and prevention remains limited. This reflects several unresolved conceptual and methodological gaps.

### RA-specific risk stratification

Existing VTE risk models are derived from general population cohorts and fail to incorporate RA-specific determinants such as disease activity, cumulative inflammatory burden, autoantibody status, disability and treatment exposure. Future work should focus on developing models that integrate traditional risk factors with time-varying disease-related variables, as static baseline assessments are unlikely to capture thrombotic risk in a disease characterized by fluctuating inflammatory activity.

### Biomarkers of inflammation-driven thrombosis

RA offers a unique model of chronic inflammation-associated thrombosis, but clinically useful biomarkers for VTE risk stratification are lacking. Numerous markers of endothelial dysfunction, coagulation activation, platelet activation and impaired fibrinolysis are elevated in RA, particularly during active disease, yet prospective data linking these markers to incident VTE are sparse. Future studies should prioritize integrated biomarker signatures rather than single markers, assess them longitudinally and evaluate their predictive value.

### Preventive strategies and trial design

There is a striking absence of randomized trials addressing VTE prevention specifically in RA outside surgical or hospitalized settings. A recent observational study by Lowell *et al.* reported that among patients with prior VTE receiving JAKi, recurrent VTE occurred only in those not receiving anticoagulation, while no recurrent events were observed during periods of concurrent anticoagulant therapy, suggesting a protective effect in this high-risk population [82]. Given the persistent prothrombotic state associated with active disease, recurrent flares and certain treatment exposures, the role of targeted or extended thromboprophylaxis remains undefined. Feasible study designs may require strategies focusing on patients with sustained high disease activity, prior VTE or multiple concurrent risk factors.

## Conclusions

RA is associated with a substantially increased risk of VTE, reflecting the interplay between chronic systemic inflammation, immune dysregulation, endothelial dysfunction and hypercoagulability. Thrombotic risk in RA is dynamic and heterogeneous, varying with disease activity, disease duration, comorbidities and treatment exposure, and remains elevated even during clinical remission.

Current approaches to VTE risk assessment rely on models developed for the general population and do not account for RA-specific determinants of risk. Effective control of inflammatory disease activity is central to VTE prevention, yet the absence of RA-specific risk stratification tools, validated biomarkers and preventive trials represents a major unmet need.

Advancing towards precision-based VTE prevention in RA will require integrated epidemiological, mechanistic and interventional research.

## Data Availability

No new data were generated or analysed in support of this article.

## References

[1] Fazal ZA , Avina-Galindo AM, Marozoff S et al Risk of venous thromboembolism in patients with rheumatoid arthritis: a meta-analysis of observational studies. BMC Rheumatol 2024;8:5. 38308337 10.1186/s41927-024-00376-9 PMC10836002

[2] Semb AG , Ikdahl E, Wibetoe G, Crowson C, Rollefstad S. Atherosclerotic cardiovascular disease prevention in rheumatoid arthritis. Nat Rev Rheumatol 2020;16:361–79. 32494054 10.1038/s41584-020-0428-y

[3] Holmqvist ME , Neovius M, Eriksson J et al Risk of venous thromboembolism in patients with rheumatoid arthritis and association with disease duration and hospitalization. JAMA 2012;308:1350–6. 23032551 10.1001/2012.jama.11741

[4] Kim SC , Schneeweiss S, Liu J, Solomon DH. The risk of venous thromboembolism in patients with rheumatoid arthritis. Arthritis Care Res 2013;65:7. 10.1002/acr.22039 PMC4090802 23666917

[5] Choi HK , Rho YH, Zhu Y et al The risk of pulmonary embolism and deep vein thrombosis in rheumatoid arthritis: a UK population-based outpatient cohort study. Ann Rheum Dis 2013;72:1182–7. 22930596 10.1136/annrheumdis-2012-201669

[6] Ogdie A , Kay McGill N, Shin DB et al Risk of venous thromboembolism in patients with psoriatic arthritis, psoriasis and rheumatoid arthritis: a general population-based cohort study. Eur Heart J 2018;39:3608–14. 28444172 10.1093/eurheartj/ehx145 PMC6186275

[7] Ketfi C , Boutigny A, Mohamedi N et al Risk of venous thromboembolism in rheumatoid arthritis. Joint Bone Spine 2021;88:105122. 33346109 10.1016/j.jbspin.2020.105122

[8] Li L , Lu N, Avina-Galindo AM et al The risk and trend of pulmonary embolism and deep vein thrombosis in rheumatoid arthritis: a general population-based study. Rheumatology (Oxford) 2021;60:188–95. 32617563 10.1093/rheumatology/keaa262 PMC7785304

[9] Russell MD , Bechman K, Gibson M et al Risk of venous thromboembolism in people with RA: a population-based study in the UK. Rheumatology (Oxford) 2025;64:6224–32. 40795208 10.1093/rheumatology/keaf430 PMC12671859

[10] Conforti A , Berardicurti O, Pavlych V et al Incidence of venous thromboembolism in rheumatoid arthritis, results from a "real-life" cohort and an appraisal of available literature. Medicine (Baltimore) 2021;100:e26953. 34414960 10.1097/MD.0000000000026953 PMC8376311

[11] Molander V , Bower H, Frisell T, Askling J. Risk of venous thromboembolism in rheumatoid arthritis, and its association with disease activity: a nationwide cohort study from Sweden. Ann Rheum Dis 2021;80:169–75. 33032998 10.1136/annrheumdis-2020-218419

[12] Kang JH , Keller JJ, Lin YK, Lin HC. A population-based case-control study on the association between rheumatoid arthritis and deep vein thrombosis. J Vasc Surg 2012;56:1642–8. 23085092 10.1016/j.jvs.2012.05.087

[13] Chung W-S , Peng C-L, Lin C-L et al Rheumatoid arthritis increases the risk of deep vein thrombosis and pulmonary thromboembolism: a nationwide cohort study. Ann Rheum Dis 2014;73:1774–80. 23926057 10.1136/annrheumdis-2013-203380

[14] Matta F , Singala R, Yaekoub AY, Najjar R, Stein PD. Risk of venous thromboembolism with rheumatoid arthritis. Thromb Haemost 2009;101:134–8. 19132199

[15] Bacani AK , Gabriel SE, Crowson CS, Heit JA, Matteson EL. Noncardiac vascular disease in rheumatoid arthritis: increase in venous thromboembolic events? Arthritis Rheum 2012;64:53–61. 21905005 10.1002/art.33322 PMC3474372

[16] Lee JJ , Pope JE. A meta-analysis of the risk of venous thromboembolism in inflammatory rheumatic diseases. Arthritis Res Ther 2014;16:435. 25253302 10.1186/s13075-014-0435-y PMC4207310

[17] Hu LJ , Ji B, Fan HX. Venous thromboembolism risk in rheumatoid arthritis patients: a systematic review and updated meta-analysis. Eur Rev Med Pharmacol Sci 2021;25:7005–13. 34859863 10.26355/eurrev_202111_27249

[18] Ojeda-Fernandez L , Calvisi S, Torrigiani G et al Incidence and risk of arterial and venous thrombotic events in systemic lupus erythematosus patients: a population-based study. Rheumatology (Oxford) 2025;64:2723–30. 39276162 10.1093/rheumatology/keae496

[19] Chen I-W , Wang W-T, Lai Y-C et al Association between systemic sclerosis and risk of cerebrovascular and cardiovascular disease: a meta-analysis. Sci Rep 2024;14:6445. 38499699 10.1038/s41598-024-57275-9 PMC10948904

[20] Roelsgaard IK , Ikdahl E, Rollefstad S et al Smoking cessation is associated with lower disease activity and predicts cardiovascular risk reduction in rheumatoid arthritis patients. Rheumatology (Oxford) 2020;59:1997–2004. 31782789 10.1093/rheumatology/kez557 PMC7382591

[21] Oudart F , Thomas M, Combier A et al Risk factors associated with venous thromboembolism in rheumatoid arthritis in clinical practice. Clin Exp Rheumatol 2025;43:1285–93. 40314997 10.55563/clinexprheumatol/7r3hxm

[22] Heit JA , Kobbervig CE, James AH et al Trends in the incidence of venous thromboembolism during pregnancy or postpartum: a 30-year population-based study. Ann Intern Med 2005;143:697–706. 16287790 10.7326/0003-4819-143-10-200511150-00006

[23] Pomp ER , Lenselink AM, Rosendaal FR, Doggen CJ. Pregnancy, the postpartum period and prothrombotic defects: risk of venous thrombosis in the MEGA study. J Thromb Haemost 2008;6:632–7. 18248600 10.1111/j.1538-7836.2008.02921.x

[24] Champion ML , Blanchard CT, Lu MY et al A more selective vs a standard risk-stratified, heparin-based, obstetric thromboprophylaxis protocol. JAMA 2024;332:310–7. 38935391 10.1001/jama.2024.8684 PMC11211987

[25] Bleau N , Patenaude V, Abenhaim HA. Risk of venous thromboembolic events in pregnant patients with autoimmune diseases: a population-based study. Clin Appl Thromb Hemost 2016;22:285–91. 25294635 10.1177/1076029614553023

[26] Aljary H , Czuzoj-Shulman N, Spence AR, Abenhaim HA. Pregnancy outcomes in women with rheumatoid arthritis: a retrospective population-based cohort study. J Matern Fetal Neonatal Med 2020;33:618–24. 30189769 10.1080/14767058.2018.1498835

[27] Barnabe C , Faris PD, Quan H. Canadian pregnancy outcomes in rheumatoid arthritis and systemic lupus erythematosus. Int J Rheumatol 2011;2011:345727. 22028718 10.1155/2011/345727 PMC3199043

[28] Bharti B , Lee SJ, Lindsay SP et al Disease severity and pregnancy outcomes in women with rheumatoid arthritis: results from the organization of teratology information specialists autoimmune diseases in pregnancy project. J Rheumatol 2015;42:1376–82. 25877497 10.3899/jrheum.140583

[29] Yoshimura M , Fujieda Y, Sugawara M et al Disease activity as a risk factor for venous thromboembolism in rheumatoid arthritis analysed using time-averaged DAS28CRP: a nested case-control study. Rheumatol Int 2022;42:1939–46. 35384451 10.1007/s00296-022-05121-4

[30] Westerlind H , Kastbom A, Rönnelid J et al The association between autoantibodies and risk for venous thromboembolic events among patients with rheumatoid arthritis. Rheumatology (Oxford) 2023;62:2106–12. 36255271 10.1093/rheumatology/keac601 PMC10234203

[31] Olech E , Merrill JT. The prevalence and clinical significance of antiphospholipid antibodies in rheumatoid arthritis. Curr Rheumatol Rep 2006;8:100–8. 16569368 10.1007/s11926-006-0049-8

[32] Erkan D. Antiphospholipid syndrome: to classify or not to classify? Turk J Haematol 2024;41:37–40. 38284227 10.4274/tjh.galenos.2024.2024.0003 PMC10918394

[33] Mameli A , Barcellona D, Marongiu F. Rheumatoid arthritis and thrombosis. Clin Exp Rheumatol 2009;27:846–55. 19917173

[34] Bordy R , Totoson P, Prati C et al Microvascular endothelial dysfunction in rheumatoid arthritis. Nat Rev Rheumatol 2018;14:404–20. 29855620 10.1038/s41584-018-0022-8

[35] Wållberg-Jonsson S , Cvetkovic JT, Sundqvist K-G et al Activation of the immune system and inflammatory activity in relation to markers of atherothrombotic disease and atherosclerosis in rheumatoid arthritis. J Rheumatol 2002;29:875–82. 12022343

[36] Fox EA , Kahn SR. The relationship between inflammation and venous thrombosis. A systematic review of clinical studies. Thromb Haemost 2005;94:362–5. 16113826 10.1160/TH05-04-0266

[37] van den Oever IA , Sattar N, Nurmohamed MT. Thromboembolic and cardiovascular risk in rheumatoid arthritis: role of the haemostatic system. Ann Rheum Dis 2014;73:954–7. 24431395 10.1136/annrheumdis-2013-204767

[38] Levi M , van der Poll T, Büller HR. Bidirectional relation between inflammation and coagulation. Circulation 2004;109:2698–704. 15184294 10.1161/01.CIR.0000131660.51520.9A

[39] Witkowski M , Landmesser U, Rauch U. Tissue factor as a link between inflammation and coagulation. Trends Cardiovasc Med 2016;26:297–303. 26877187 10.1016/j.tcm.2015.12.001

[40] Szotowski B , Antoniak S, Poller W, Schultheiss HP, Rauch U. Procoagulant soluble tissue factor is released from endothelial cells in response to inflammatory cytokines. Circ Res 2005;96:1233–9. 15920023 10.1161/01.RES.0000171805.24799.fa

[41] Santos MJ , Carmona-Fernandes D, Canhão H et al Early vascular alterations in SLE and RA patients-a step towards understanding the associated cardiovascular risk. PLoS One 2012;7:e44668. 22962622 10.1371/journal.pone.0044668 PMC3433444

[42] Peters MJ , Nurmohamed MT, van Eijk IC, Verkleij CJ, Marx PF. Thrombin-activatable fibrinolysis inhibitor and its relation with inflammation in rheumatoid arthritis. Ann Rheum Dis 2009;68:1232–3. 19525410 10.1136/ard.2008.097485

[43] Rooney T , Scherzer R, Shigenaga JK et al Levels of plasma fibrinogen are elevated in well-controlled rheumatoid arthritis. Rheumatology (Oxford) 2011;50:1458–65. 21441551 10.1093/rheumatology/ker011

[44] McEntegart A , Capell HA, Creran D et al Cardiovascular risk factors, including thrombotic variables, in a population with rheumatoid arthritis. Rheumatology (Oxford) 2001;40:640–4. 11426020 10.1093/rheumatology/40.6.640

[45] Esmon CT. Coagulation inhibitors in inflammation. Biochem Soc Trans 2005;33:401–5. 15787615 10.1042/BST0330401

[46] Zavoriti A , Miossec P. Understanding the effects of Janus kinase inhibitors on the cardiovascular system in comparison to main biological DMARDs in rheumatoid arthritis. Autoimmun Rev 2025;24:103892. 40721038 10.1016/j.autrev.2025.103892

[47] Dijkshoorn B , Hansildaar R, Vedder D et al Impaired coagulation parameters in early RA are restored by effective antirheumatic therapy: a prospective pilot study. RMD Open 2024;10:e004838. 39740931 10.1136/rmdopen-2024-004838 PMC11748942

[48] Boilard E , Nigrovic PA, Larabee K et al Platelets amplify inflammation in arthritis via collagen-dependent microparticle production. Science 2010;327:580–3. 20110505 10.1126/science.1181928 PMC2927861

[49] Knijff-Dutmer EA , Koerts J, Nieuwland R, Kalsbeek-Batenburg EM, van de Laar MA. Elevated levels of platelet microparticles are associated with disease activity in rheumatoid arthritis. Arthritis Rheum 2002;46:1498–503. 12115179 10.1002/art.10312

[50] Stojanovic A , Veselinovic M, Zong Y et al Increased expression of extracellular vesicles is associated with the procoagulant state in patients with established rheumatoid arthritis. Front Immunol 2021;12:718845. 34394126 10.3389/fimmu.2021.718845 PMC8358654

[51] Abebaw D , Akelew Y, Adugna A et al Extracellular vesicles: immunomodulation, diagnosis, and promising therapeutic roles for rheumatoid arthritis. Front Immunol 2024;15:1499929. 39624102 10.3389/fimmu.2024.1499929 PMC11609219

[52] Zhang B , Zhao M, Lu Q et al Extracellular vesicles in rheumatoid arthritis and systemic lupus erythematosus: nctions and applications. Front Immunol 2020;11:575712. 33519800 10.3389/fimmu.2020.575712 PMC7841259

[53] Chen X. Rac1 regulates platelet microparticles formation and rheumatoid arthritis deterioration. Platelets 2020;31:112–9. 30880553 10.1080/09537104.2019.1584669

[54] Muttiah B , Ng SL, Lokanathan Y, Ng MH, Law JX. Beyond blood clotting: the many roles of platelet-derived extracellular vesicles. Biomedicines 2024;12:1850. 39200314 10.3390/biomedicines12081850 PMC11351396

[55] Tessandier N , Melki I, Cloutier N et al Platelets disseminate extracellular vesicles in lymph in rheumatoid arthritis. Arterioscler Thromb Vasc Biol 2020;40:929–42. 32102567 10.1161/ATVBAHA.119.313698 PMC8073225

[56] Johannesdottir SA , Horváth-Puhó E, Dekkers OM et al Use of glucocorticoids and risk of venous thromboembolism: a nationwide population-based case-control study. JAMA Intern Med 2013;173:743–52. 23546607 10.1001/jamainternmed.2013.122

[57] Ozen G , Pedro S, Schumacher R, Simon T, Michaud K. Risk factors for venous thromboembolism and atherosclerotic cardiovascular disease: do they differ in patients with rheumatoid arthritis? RMD Open 2021;7:e001618. 34193517 10.1136/rmdopen-2021-001618 PMC8246357

[58] Prescribing information. Silver Spring, MD: US Food and Drug Administration, 2025 June 4. https://www.accessdata.fda.gov (3 January 2026, date last accessed).

[59] Atzeni F , Rodríguez-Carrio J, Popa CD et al Cardiovascular effects of approved drugs for rheumatoid arthritis. Nat Rev Rheumatol 2021;17:270–90. 33833437 10.1038/s41584-021-00593-3

[60] Xiao R , Tang P, Zhou J et al Safety of TNF-α inhibitors therapy in patients with rheumatoid arthritis: an umbrella review. EClinicalMedicine 2025;88:103488. 41181844 10.1016/j.eclinm.2025.103488 PMC12572804

[61] Chen CP , Kung PT, Chou WY, Tsai WC. Effect of introducing biologics to patients with rheumatoid arthritis on the risk of venous thromboembolism: a nationwide cohort study. Sci Rep 2021;11:17009. 34426637 10.1038/s41598-021-96508-z PMC8382764

[62] de Queiroz MJ , de Castro CT, Albuquerque FC et al Safety of biological therapy in patients with rheumatoid arthritis in administrative health databases: a systematic review and meta-analysis. Front Pharmacol 2022;13:928471. 36034855 10.3389/fphar.2022.928471 PMC9407686

[63] Jin Y , Liu J, Desai RJ. Comparative safety of biologic and targeted-synthetic DMARDs in patients with rheumatoid arthritis: a multi-database real-world cohort study. Pharmacoepidemiol Drug Saf 2025;34:e70193. 40717043 10.1002/pds.70193

[64] Winthrop KL , Saag K, Cascino MD et al Long-term safety of rituximab in rheumatoid arthritis: analysis from the SUNSTONE registry. Arthritis Care Res (Hoboken) 2018;71:993–1003. 30295434 10.1002/acr.23781 PMC6806017

[65] Curtis JR , Perez-Gutthann S, Suissa S et al; Actemra Pharmacoepidemiology Board. Tocilizumab in rheumatoid arthritis: a case study of safety evaluations of a large postmarketing data set from multiple data sources. Semin Arthritis Rheum 2015;44:381–8. 25300699 10.1016/j.semarthrit.2014.07.006

[66] Ascef BDO , Almeida MO, Medeiros-Ribeiro ACD et al Therapeutic equivalence of biosimilar and reference biologic drugs in rheumatoid arthritis: a systematic review and meta-analysis. JAMA Netw Open 2023;6:e2315872. 37234004 10.1001/jamanetworkopen.2023.15872 PMC10220520

[67] Desai RJ , Pawar A, Weinblatt ME, Kim SC. Comparative risk of venous thromboembolism in rheumatoid arthritis patients receiving tofacitinib versus those receiving tumor necrosis factor inhibitors: an observational cohort study. Arthritis Rheumatol 2019;71:892–900. 30552833 10.1002/art.40798

[68] Tanaka Y , Luo Y, O’Shea JJ, Nakayamada S. Janus kinase-targeting therapies in rheumatology: a mechanisms-based approach. Nat Rev Rheumatol 2022;18:133–45. 34987201 10.1038/s41584-021-00726-8 PMC8730299

[69] Ozdede A , Yazıcı H. Cardiovascular and cancer risk with tofacitinib in rheumatoid arthritis. N Engl J Med 2022;386:1766. 10.1056/NEJMc2202778 35507490

[70] Hoisnard L , Pina Vegas L, Dray-Spira R et al Risk of major adverse cardiovascular and venous thromboembolism events in patients with rheumatoid arthritis exposed to JAK inhibitors versus adalimumab: a nationwide cohort study. Ann Rheum Dis 2023;82:182–8. 36198438 10.1136/ard-2022-222824

[71] Song Y-J , Cho S-K, Kim J-Y et al Risk of venous thromboembolism in Korean patients with rheumatoid arthritis treated with Janus kinase inhibitors: a nationwide population-based study. Semin Arthritis Rheum 2023;61:152214. 37172496 10.1016/j.semarthrit.2023.152214

[72] Kremer JM , Bingham CO, Cappelli LC et al Postapproval comparative safety study of tofacitinib and biological disease-modifying antirheumatic drugs: 5-year results from a United States-Based Rheumatoid Arthritis Registry. ACR Open Rheumatol 2021;3:173–84. 33570260 10.1002/acr2.11232 PMC7966883

[73] Salinas CA , Louder A, Polinski J et al; B023 Study Consortium. Evaluation of VTE, MACE, and serious infections among patients with RA treated with baricitinib compared to TNFi: a multi-database study of patients in routine care using disease registries and claims databases. Rheumatol Ther 2023;10:201–23. 36371760 10.1007/s40744-022-00505-1 PMC9660195

[74] Molander V , Bower H, Frisell T et al; ARTIS study group. Venous thromboembolism with JAK inhibitors and other immune-modulatory drugs: a Swedish comparative safety study among patients with rheumatoid arthritis. Ann Rheum Dis 2023;82:189–97. 36150749 10.1136/ard-2022-223050 PMC9887398

[75] Ytterberg SR , Bhatt DL, Mikuls TR et al; ORAL Surveillance Investigators. Cardiovascular and cancer risk with tofacitinib in rheumatoid arthritis. N Engl J Med 2022;386:316–26. 35081280 10.1056/NEJMoa2109927

[76] Granziera S , Cohen AT. VTE primary prevention, including hospitalised medical and orthopaedic surgical patients. Thromb Haemost 2015;113:1216–23. 25809340 10.1160/TH14-10-0823

[77] Henke PK , Kahn SR, Pannucci CJ et al; American Heart Association Advocacy Coordinating Committee. Call to action to prevent venous thromboembolism in hospitalized patients: a policy statement from the American Heart Association. Circulation 2020;141:e914–31. 32375490 10.1161/CIR.0000000000000769

[78] van Heereveld HA , Laan RF, van den Hoogen FH et al Prevention of symptomatic thrombosis with short term (low molecular weight) heparin in patients with rheumatoid arthritis after hip or knee replacement. Ann Rheum Dis 2001;60:974–6. 11557656 10.1136/ard.60.10.974 PMC1753387

[79] Khan F , Tritschler T, Kahn SR, Rodger MA. Venous thromboembolism. Lancet 2021;398:64–77. 33984268 10.1016/S0140-6736(20)32658-1

[80] Konstantinides SV , Meyer G. The 2019 ESC guidelines on the diagnosis and management of acute pulmonary embolism. Eur Heart J 2019;40:3453–5. 31697840 10.1093/eurheartj/ehz726

[81] Charles-Schoeman C , Fleischmann R, Mysler E et al Risk of venous thromboembolism with tofacitinib versus tumor necrosis factor inhibitors in cardiovascular risk-enriched rheumatoid arthritis patients. Arthritis Rheumatol 2024;76:1218–29. 38481002 10.1002/art.42846

[82] Lowell JA , Sharma G, Swaminath A, Sultan K. Pharmacologic anticoagulation is associated with a lower risk of recurrent venous thromboembolic events during Janus kinase inhibitor use for patients with a prior thrombosis. Inflamm Bowel Dis 2025;31:725–32. 38704439 10.1093/ibd/izae100

